# A Hybrid Model for Copper Futures Price Forecasting Utilizing Complexity-Aware Variational Mode Decomposition and Reconstruction and Multi-Behavior-Triggered Interaction Modeling

**DOI:** 10.3390/e28030320

**Published:** 2026-03-12

**Authors:** Yan Li, Dezhi Liu

**Affiliations:** 1 School of Finance, Anhui Sanlian University, Hefei 230601, China; ly@mail.slu.edu.cn; 2 School of Internet, Anhui University, Hefei 230601, China

**Keywords:** copper future, time-series decomposition, volatility- and behavior-aware Reversible Normalization, graph neural network, market signal modeling

## Abstract

Accurate forecasting of copper futures prices is crucial for risk management and investment decisions. However, existing approaches primarily rely on historical prices and incorporate behavioral signals without a unified modeling framework. To address this limitation, we propose MBTI-Net (Multi-source Behavior-Triggered Interaction Network), a behavior-aware forecasting framework for heterogeneous copper market data. We first construct a compact behavioral factor from Baidu search indices via a multi-view projection strategy that preserves structural and predictive information. We then develop a complexity-aware reconstruction mechanism that aggregates intrinsic mode functions into multi-frequency components based on fuzzy entropy and energy. To accommodate distributional and volatility differences between behavioral and market variables, we introduce VB-ReVIN (Volatility- and Behavior-aware Reversible Instance Normalization). Building upon these representations, MBTI-Net models dynamic multi-source interactions triggered by behavioral intensity and market conditions, enabling adaptive cross-source information fusion. Experiments on LME and SHFE copper futures datasets demonstrate consistent improvements over state-of-the-art benchmarks, highlighting the importance of explicitly modeling behavior-driven dependencies in financial forecasting.

## 1. Introduction

### 1.1. Background

Copper plays a foundational role in the global energy transition. As electrification accelerates and renewable energy capacity expands, demand for copper-intensive infrastructure—such as power grids, electric vehicles, wind turbines, and photovoltaic systems—has risen substantially. Owing to its superior electrical and thermal conductivity, copper is indispensable in energy transmission, storage, and efficiency-enhancing technologies [1,2,3]. This structural reconfiguration of demand has reshaped copper market dynamics. Beyond traditional industrial cycles, prices are increasingly influenced by energy transition policies, technological adoption, global supply constraints, and capital flows, leading to elevated volatility and heightened sensitivity to macroeconomic and geopolitical shocks.

Accurate forecasting is therefore economically and strategically critical. Reliable price projections support risk management, investment planning, and policy assessment, particularly in the context of large-scale decarbonization efforts [4]. Given copper’s close linkage to manufacturing activity and clean energy deployment, its price movements are often interpreted as forward-looking indicators of economic momentum [5,6]. Importantly, the information environment surrounding commodity markets has evolved substantially, with the faster dissemination and greater diversity of information being incorporated into prices. The expansion of digital platforms has generated high-frequency behavioral signals—such as online search intensity—that reflect market expectations and risk perceptions in real time. These attention-based measures, compared with traditional macroeconomic indicators released with a delay, respond immediately to policy shifts, supply disruptions, and technological developments. Emerging evidence in financial economics suggests that such behavioral proxies can serve as leading indicators, especially in markets undergoing significant uncertainty and structural transition [7,8,9]. For copper markets, which are in a state of transformation, integrating these behavioral signals could significantly enhance forecasting performance by capturing short-term dynamics and evolving sentiment. Given the different determinants operating across time horizons, this study concentrates on short-term daily forecasting, where high-frequency behavioral responses and information diffusion play a more dominant role than long-run structural forces.

In this study, we focus on short-term forecasting of daily copper futures closing prices. Although the prediction target is a univariate time series, its short-run evolution reflects the aggregated influence of heterogeneous financial variables, investor reactions, and rapidly changing market attention. In high-frequency settings, online search intensity serves as a timely proxy for evolving expectations and sentiment, which may not be fully captured by traditional macroeconomic indicators. Therefore, effectively modeling cross-source interactions and multi-scale temporal structures is essential for improving short-term forecasting accuracy.

### 1.2. Research Gaps

Despite growing interest in incorporating behavioral signals into financial forecasting, several methodological limitations remain.

First, existing approaches typically model behavioral time series either directly or through loosely integrated feature extraction modules, without explicitly disentangling structural dynamics across temporal scales. Moreover, they often assume uniform temporal contribution, overlooking the heterogeneous informational relevance of different time periods. Consequently, frequency-specific patterns and behavior-sensitive temporal segments may not be adequately captured.

Second, while behavioral indicators are increasingly used as supplementary predictors, most forecasting frameworks primarily model temporal dependencies within individual modalities. Explicit mechanisms for capturing cross-modal structural interactions between behavioral attention and financial factors remain limited. As a result, the dynamic ways in which behavioral signals may strengthen or weaken financial factor relationships are seldom modeled directly.

Third, although normalization techniques such as ReVIN [10] address non-stationarity and distribution shift, they are largely developed for single-source settings. In multi-source environments where behavioral and financial variables exhibit distinct statistical characteristics and volatility regimes, conventional normalization strategies do not explicitly account for cross-source heterogeneity, potentially leading to suboptimal distribution alignment.

### 1.3. Main Contributions

To address these limitations, this study proposes a unified forecasting framework centered on a core methodological innovation: a behavior-triggered factor interaction mechanism. Two targeted methodological enhancements are further incorporated to strengthen signal representation and distributional alignment within this framework.

The main contributions of this work are summarized as follows:

1. We introduce a behavior-triggered factor interaction mechanism that dynamically reshapes cross-factor dependencies through behavior-conditioned structural modulation. This mechanism is operationalized via similarity-based graph construction and mix-hop graph propagation, enabling behavioral signals to regulate the intensity and configuration of financial factor relationships rather than serving as passive inputs.
2. We extend the ReVIN framework by incorporating regime-level volatility information and separating behavior-driven and market-driven components. The resulting VB-ReVIN performs adaptive group- and regime-dependent affine transformations, improving distributional alignment across heterogeneous multi-source data.
3. We design a structured multi-scale behavioral representation strategy that leverages an improved VMD-based decomposition to separate high- and low-frequency components, together with a time-step attention mechanism to adaptively weight temporal observations.
4. Extensive experiments on copper futures markets demonstrate consistent performance improvements over competitive baselines.

### 1.4. Organization and Structure

The remainder of this paper is organized as follows: Section 2 reviews the related literature and summarizes existing work. Section 3 presents the proposed framework and describes the corresponding methodology in detail. Section 4 reports the experimental setup and discusses the empirical results. Finally, Section 5 concludes the paper by highlighting the main findings and outlining directions for future research.

## 2. Related Works

This section reviews existing studies on copper futures price forecasting from two perspectives: (1) data sources and (2) forecasting models.

### 2.1. Data Sources for Copper Futures Price Forecasting

Copper futures prices are influenced by multiple factors; therefore, their prediction relies on diverse data sources, including both historical price data and combined data sources. Historical price data provide insight into copper futures price dynamics over time and have been widely applied to extract trend-related information for forecasting copper futures prices. For instance, in ref. [11], daily copper prices were predicted based on the stability of historical series. In ref. [12], time-domain features of historical prices were incorporated to forecast monthly copper futures prices, while in ref. [13], seasonal characteristics of historical copper data were utilized for daily price prediction. Similarly, in ref. [14], historical copper futures price returns were considered to predict monthly prices.

Recently, combined data sources have attracted increasing attention because historical prices alone cannot sufficiently capture market fluctuations in copper futures. Combined data sources integrate price information with other relevant factors, including macroeconomic indicators such as energy prices (e.g., crude oil, natural gas, and coal), prices of other metal futures (e.g., gold, silver, and iron), and additional related indicators [15,16,17].

Moreover, search engine data have emerged as a valuable supplementary data source, as they reflect users’ behavioral intentions and interests through search activity. Given this characteristic, search trends related to copper futures can provide timely insights into market sentiment and evolving short-term market conditions. Such behavioral signals are particularly useful for short-horizon forecasting, where price fluctuations are often driven by investor reactions, information diffusion, and speculative dynamics. However, only a limited number of studies have explored the integration of search engine data into copper futures price forecasting.

### 2.2. Copper Futures Price Forecasting Models

Forecasting models applied to copper futures price prediction mainly consist of time-series models and artificial intelligence (AI) approaches. Traditional time-series models such as VAR (vector autoregression), ARIMA (autoregressive integrated moving average), GARCH (generalized autoregressive conditional heteroskedasticity), and their variants rely primarily on historical dynamics to forecast future prices [2,11,18]. Although multivariate frameworks such as VAR can incorporate multiple endogenous variables and even exogenous extensions, these models are typically built upon linear specifications. As a result, they may face limitations in capturing complex nonlinear interactions, structural shifts, and high-dimensional cross-source dependencies. As noted earlier, copper futures prices are influenced by diverse and potentially nonlinear factors, which may reduce the effectiveness of purely linear modeling approaches [19]. Time-series models also encounter difficulties when handling large-scale datasets with intricate nonlinear dependencies.

To overcome these limitations, numerous studies have proposed AI-based models, including support vector machines (SVMs), artificial neural networks (ANNs), long short-term memory networks (LSTMs), gated recurrent units (GRUs), and convolutional neural networks (CNNs). These methods have shown strong capabilities in learning from nonlinear patterns and high-frequency data, which are often observed in financial time series [15,20,21]. However, even among these models, certain limitations persist. For example, ANNs often fail to retain temporal dependencies over long sequences, while CNNs, though effective in capturing spatial features, are less suited for modeling time-dependent dynamics. Recurrent models like LSTM and GRU, while more effective for sequence data, may still suffer from degraded performance in long-horizon forecasting due to their step-by-step processing nature [22,23].

More recently, transformer-based architectures have emerged as a powerful alternative for time-series forecasting. By leveraging self-attention mechanisms, transformer models enable parallel computation and more effective modeling of long-range dependencies compared with recurrent networks. Variants such as Informer [24], Autoformer [25], DTSformer [26], and FEDformer [27] have demonstrated strong performance in long-horizon prediction. However, despite their advantages in temporal modeling, most transformer-based approaches primarily focus on capturing intra-series dependencies within a single data modality. In multi-source forecasting contexts, auxiliary variables such as behavioral indicators are typically incorporated through feature concatenation or simple embedding strategies, without explicitly modeling cross-modal structural interactions.

Furthermore, recent advancements have introduced hybrid models that combine deep learning architectures with optimization techniques or signal decomposition methods (e.g., wavelet transform, variational mode decomposition (VMD)). These approaches aim to enhance forecasting performance by capturing multi-scale characteristics and tuning model hyperparameters more effectively [28,29,30]. A categorized summary of major copper futures price forecasting techniques and representative studies is presented in Table 1.

## 3. Methodology

### 3.1. Overall Workflow Overview

**Figure 1:**
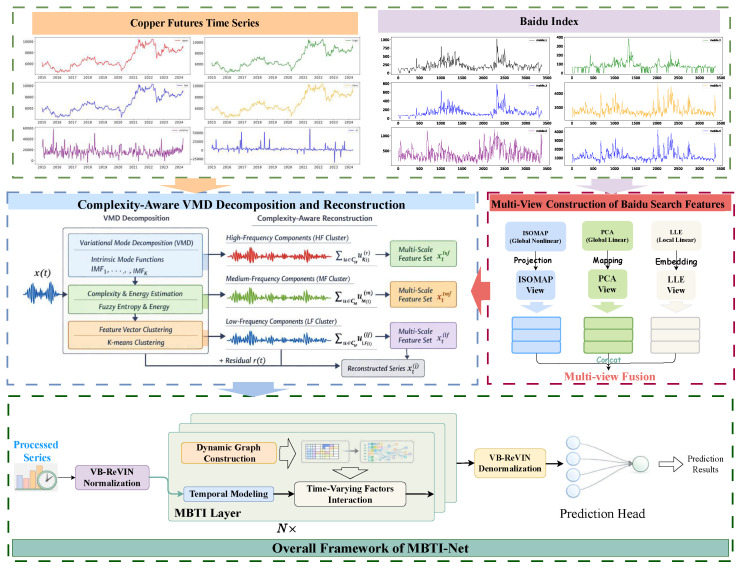
Overview of data characteristics and the proposed MBTI-Net framework. **Top**: Copper futures price series (2257 daily observations) and representative Baidu search index series. **Middle**: Complexity-aware VMD decomposition and clustering into three bands (HF, MF, LF) based on fuzzy entropy and energy. **Bottom**: The proposed MBTI-Net deep learning architecture for dynamic graph-based temporal modeling and prediction.

As illustrated in Figure 1, the proposed framework integrates multi-source copper-related data into a unified forecasting pipeline. Let $x_{t}^{\mathrm{raw}}\in R^{C_{0}}$ denote the raw feature vector at time *t*, consisting of a market group $G_{\mathrm{mkt}}$ (e.g., copper futures prices, volumes, and technical indicators) and a behavioral group $G_{\mathrm{beh}}$ derived from Baidu search activity.

The workflow consists of four main stages. First, a multi-view feature construction step transforms the raw Baidu Index into compact behavioral representations, yielding the behavioral vector $z_{t}^{\mathrm{BI}}$ and forming $x_{t}^{\mathrm{raw}}=\left[x_{t}^{\mathrm{mkt},\mathrm{raw}};z_{t}^{\mathrm{BI}}\right]$. Second, a complexity-aware VMD module decomposes each raw feature into multi-scale components and reconstructs scale-specific features ${\left\{x_{t}^{\left(s\right)}\right\}}_{s\in \{\mathrm{hf},\mathrm{mf},\mathrm{lf}\}}$. Third, a Volatility- and Behavior-Aware reversible normalization (VB-ReVIN) module standardizes the multi-scale features under volatility regimes to obtain normalized representations $\left\{{\hat{x}}_{t}^{\left(s\right)}\right\}$ while preserving invertibility. Finally, stacked Multi-Behavior-Triggered Interaction (MBTI) layers model temporal dependencies and time-varying cross-factor interactions based on ${\hat{x}}_{t}=\left[{\hat{x}}_{t}^{\left(\mathrm{hf}\right)};{\hat{x}}_{t}^{\left(\mathrm{mf}\right)};{\hat{x}}_{t}^{\left(\mathrm{lf}\right)}\right]$, followed by a prediction head that outputs the copper futures price forecast.

The detailed formulation of each component is presented in the following subsections.

### 3.2. Multi-View Construction of Baidu Search Features

To mitigate noise and multicollinearity in the Baidu Index data, we adopt a multi-view feature extraction strategy following [36].

Let $b_{t}^{\mathrm{raw}}\in R^{M}$ denote the raw Baidu search vector at time *t*, where *M* is the number of selected copper-related keywords. We first apply a logarithmic transformation element-wise to stabilize variance: ${\tilde{b}}_{t}=\log\left(1+b_{t}^{\mathrm{raw}}\right).$

Three complementary low-dimensional representations are then extracted: (i) a linear global embedding via principal component analysis (PCA) [37], (ii) a nonlinear local embedding via locally linear embedding (LLE) [38], and (iii) a nonlinear global embedding via Isometric Mapping (ISOMAP) [39].

Let $z_{t}^{\mathrm{PCA}}$, $z_{t}^{\mathrm{LLE}}$, and $z_{t}^{\mathrm{ISO}}$ denote the corresponding embeddings. The final behavioral feature vector $z_{t}^{\mathrm{BI}}$ is obtained by concatenation, as follows:

(1) $$z_{t}^{\mathrm{BI}}=\left[z_{t}^{\mathrm{PCA}};z_{t}^{\mathrm{LLE}};z_{t}^{\mathrm{ISO}}\right]\in R^{C_{\mathrm{beh}}}.$$

This compact representation replaces the raw behavioral block in $x_{t}^{\mathrm{raw}}$ and serves as the behavioral input to the subsequent multi-scale decomposition module.

### 3.3. Complexity-Aware Variational Mode Decomposition and Reconstruction

Directly modeling raw copper futures and related variables may obscure heterogeneous dynamics operating at different temporal scales. In futures markets, high-frequency fluctuations often reflect speculative trading and short-term sentiment shocks, whereas low-frequency components capture persistent macroeconomic trends. To disentangle these scale-specific dynamics, we apply VMD [40] to each raw feature dimension.

**Variational mode decomposition.** Let $x_{t,c}^{\mathrm{raw}}$ denote the *c*-th raw feature at time *t*, where $c\in \{1,\ldots ,C_{0}\}$. VMD decomposes the univariate series ${\left\{x_{t,c}^{\mathrm{raw}}\right\}}_{t=1}^{T}$ into *K* intrinsic mode functions (IMFs) ${\left\{u_{k,c}\left(t\right)\right\}}_{k=1}^{K}$, each associated with a narrow-band frequency component. After convergence, the original series is reconstructed as

(2) $$x_{t,c}^{\mathrm{raw}}=\sum_{k=1}^{K}u_{k,c}\left(t\right)+r_{c}\left(t\right),$$

where $r_{c}\left(t\right)$ denotes a small residual term.

**Complexity-aware grouping.** Instead of grouping IMFs by frequency index, we characterize each component using: (i) fuzzy entropy (FEn), which measures temporal irregularity, and (ii) energy $E_{k,c}=\sum_{t=1}^{T}{\left(u_{k,c}\left(t\right)\right)}^{2}$, reflecting amplitude magnitude.

Each IMF is summarized by the two-dimensional descriptor

(3) $$v_{k,c}={\left[\mathrm{FEn}\left(u_{k,c}\right),\log\left(1+E_{k,c}\right)\right]}^{⊤}.$$

For each feature *c*, the *K* IMFs are clustered via K-means into three groups ordered by average entropy and labeled as high-frequency (HF), medium-frequency (MF), and low-frequency (LF). Let $C_{\mathrm{HF},c}$, $C_{\mathrm{MF},c}$, and $C_{\mathrm{LF},c}$ denote the corresponding index sets. The reconstructed scale-specific subseries are defined as

(4) $$x_{t,c}^{\left(s\right)}=\sum_{k\in C_{s,c}}u_{k,c}\left(t\right),\;s\in \left\{\mathrm{hf},\mathrm{mf},\mathrm{lf}\right\}.$$

Finally, concatenating all feature dimensions at each scale yields the multi-scale representations $x_{t}^{\left(s\right)}={\left[x_{t,1}^{\left(s\right)},\ldots ,x_{t,C_{0}}^{\left(s\right)}\right]}^{⊤}\in R^{C_{0}},\;s\in \left\{\mathrm{hf},\mathrm{mf},\mathrm{lf}\right\}.$

### 3.4. Volatility- and Behavior-Aware Reversible Normalization

**Figure 2:**
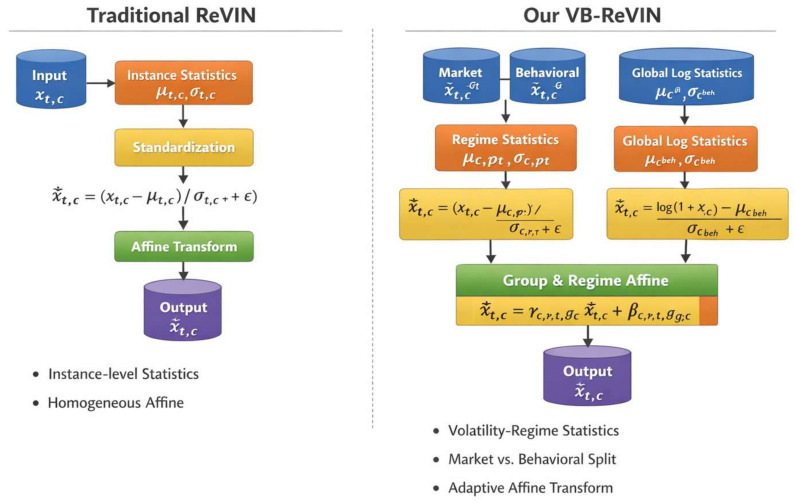
Comparison between traditional ReVIN and our proposed VB-ReVIN.

Market variables and behavioral signals exhibit heterogeneous responses across volatility regimes, resulting in regime-dependent distribution shifts. As shown in Figure 2, to address these shifts while maintaining the reversibility of ReVIN [10], we develop VB-ReVIN. The proposed module normalizes market features using regime-specific statistics and behavioral features through group-wise normalization. As previously defined, the raw features are partitioned into a market group $G_{\mathrm{mkt}}$ and a behavioral group $G_{\mathrm{beh}}$. After complexity-aware VMD reconstruction, each feature dimension *c* is represented at multiple scales $x_{t,c}^{\left(s\right)}$, s∈{hf,mf,lf}.

**Volatility regime identification.** The volatility regime is determined from the raw settlement price series $p_{t}^{\mathrm{raw}}$. Let $r_{t}=\log p_{t}^{\mathrm{raw}}-\log p_{t-1}^{\mathrm{raw}}$ denote the log-return. Local volatility is estimated by $\sigma_{t}^{\mathrm{loc}}=\sqrt{\frac{1}{w}\sum_{i=t-w+1}^{t}r_{i}^{2}}.$ Time steps are discretized into three regimes $\rho_{t}\in \left\{\mathrm{low},\mathrm{mid},\mathrm{high}\right\}$ based on empirical quantiles of $\sigma_{t}^{\mathrm{loc}}$.

**Regime-aware standardization.** For each scale *s* and market feature $c\in G_{\mathrm{mkt}}$, we compute regime-specific statistics $\mu_{c,r}^{\left(s\right)},\;\sigma_{c,r}^{\left(s\right)}$ over $T_{r}=\left\{t:\rho_{t}=r\right\}$. Standardization is performed as ${\tilde{x}}_{t,c}^{\left(s\right)}=\frac{x_{t,c}^{\left(s\right)}-\mu_{c,\rho_{t}}^{\left(s\right)}}{\sigma_{c,\rho_{t}}^{\left(s\right)}+\epsilon }.$

For behavioral features $c\in G_{\mathrm{beh}}$, global scale-wise statistics $\mu_{c}^{(s),\mathrm{beh}}$ and $\sigma_{c}^{(s),\mathrm{beh}}$ are used: ${\tilde{x}}_{t,c}^{\left(s\right)}=\frac{x_{t,c}^{\left(s\right)}-\mu_{c}^{(s),\mathrm{beh}}}{\sigma_{c}^{(s),\mathrm{beh}}+\epsilon }.$

**Reversible affine transformation.** We further apply a learnable group- and regime-conditioned affine mapping, as follows:

(5) $${\hat{x}}_{t,c}^{\left(s\right)}=\gamma_{c,\rho_{t},g_{c}}^{\left(s\right)}{\tilde{x}}_{t,c}^{\left(s\right)}+\beta_{c,\rho_{t},g_{c}}^{\left(s\right)},$$

where $g_{c}\in \left\{\mathrm{mkt},\mathrm{beh}\right\}$.

During inference, the transformation is inverted by ${\tilde{x}}_{t,c}^{\left(s\right)}=\frac{{\hat{x}}_{t,c}^{\left(s\right)}-\beta_{c,\rho_{t},g_{c}}^{\left(s\right)}}{\gamma_{c,\rho_{t},g_{c}}^{\left(s\right)}},$ followed by de-standardization using the corresponding statistics. This preserves the invertibility property central to ReVIN.

VB-ReVIN therefore adapts normalization to both volatility regimes and feature groups while maintaining strict reversibility.

### 3.5. Multi-Behavior-Triggered Interaction Layer

Copper futures dynamics are shaped by complex interactions among prices, volumes, steel futures, and multi-scale behavioral indicators. These interactions are inherently time-varying: short-term shocks propagate rapidly through speculative channels, while long-term movements reflect persistent macroeconomic and sentiment-driven trends. To capture such evolving cross-factor dependencies, we design a Multi-Behavior-Triggered Interaction (MBTI) layer that integrates temporal attention with dynamic graph learning.


** **

Multi-resolution temporal encoding.

After multi-view Baidu feature construction, complexity-aware VMD decomposition, and VB-ReVIN normalization, each time step *t* is represented by three scale-specific feature vectors ${\hat{x}}_{t}^{\mathrm{hf}},{\hat{x}}_{t}^{\mathrm{mf}},{\hat{x}}_{t}^{\mathrm{lf}}\in R^{C_{0}}.$ These are concatenated as ${\hat{x}}_{t}\in R^{3C_{0}},$ and stacked to form $\hat{X}={\left[{\hat{x}}_{1},\ldots ,{\hat{x}}_{T}\right]}^{⊤}\in R^{T\times 3C_{0}}.$

**Figure 3:**
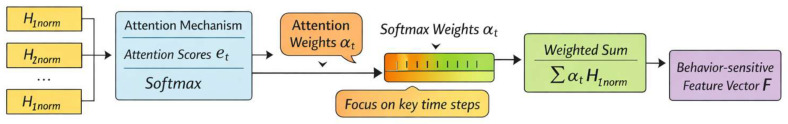
Illustration of the temporal attention mechanism for extracting behavior-sensitive representations.

The sequence is encoded using an LSTM, producing hidden states $H_{t}\in R^{d}$. As shown in Figure 3, to emphasize behavior-sensitive periods, we apply a temporal attention mechanism, as follows:

(6) $$\begin{array}{cc} e_{t} & =u^{⊤}\tanh\left(W_{h}H_{t}^{\mathrm{norm}}\right),\\ \alpha_{t} & =\frac{\exp(e_{t})}{\sum_{j=1}^{T}\exp\left(e_{j}\right)},\\ F & =\sum_{t=1}^{T}\alpha_{t}H_{t}^{\mathrm{norm}}, \end{array}$$

where $F\in R^{d}$ denotes a behavior-aware global context vector summarizing informative temporal segments.


** **

Behavior-triggered dynamic graph construction.

Financial factor interactions are not static but vary with behavioral intensity and market regimes. To model such adaptive connectivity, we generate node-level embeddings from the global context vector through a learnable projection, as follows:

(7) $$Z=\mathrm{Reshape}(FW_{p}),$$

where $W_{p}$ is trainable and $Z\in R^{N\times d_{n}}$ represents behavior-modulated embeddings of *N* factors.

A dynamic adjacency matrix is then constructed via similarity-based learning, as follows:

(8) $$A^{\mathrm{dyn}}=\mathrm{softmax}\left(\mathrm{ReLU}(ZZ^{⊤})\right),$$

where the row-wise softmax ensures normalized influence weights. This mechanism allows global behavioral signals to regulate cross-factor interaction strengths in a data-driven manner.


** **

Mix-hop interaction propagation.

Given node embeddings Z and adjacency $A^{\mathrm{dyn}}$, we initialize $H^{\left(0\right)}=Z$ and perform mix-hop propagation, as follows:

(9) $$H^{\left(l+1\right)}=\sigma \left(\sum_{k=0}^{K_{\max}}\alpha_{k}\;A^{k}H^{\left(l\right)}W^{\left(l\right)}\right),$$

where $\left\{\alpha_{k}\right\}$ controls *k*-hop neighborhood influence, $W^{\left(l\right)}$ are trainable parameters, and σ(·) denotes nonlinear activation. The resulting representation $H^{\left(L\right)}\in R^{N\times d_{n}}$ captures behavior-aware multi-order factor interactions.


** **

Factor-level self-attention refinement.

To further enhance flexibility beyond topology-driven propagation, we apply multi-head self-attention over factor representations, as follows:

(10) $$\tilde{H}=\mathrm{LayerNorm}\left(H^{\left(L\right)}+\mathrm{MHA}\left(H^{\left(L\right)},H^{\left(L\right)},H^{\left(L\right)}\right)\right),$$

where MHA(·) denotes multi-head attention. This stage enables adaptive reweighting of factor dependencies beyond the learned adjacency structure.


** **

Prediction.

The refined factor representations are pooled and passed to the prediction head, as follows:

(11) $${\hat{y}}_{t+\tau }=f_{\mathrm{pred}}\left(\mathrm{Pool}(\tilde{H})\right).$$

Residual connections are incorporated across MBTI layers to facilitate stable optimization.

Finally, outputs are restored to the original price domain via the inverse VB-ReVIN transformation.

### 3.6. Computational Complexity and Scalability

We distinguish between *offline preprocessing* and *online model computation*. Multi-view construction and VMD decomposition are performed once and cached, whereas VB-ReVIN, temporal encoding, and dynamic factor interaction are executed during training and inference.


** **

Offline preprocessing.

For sequence length *T*, VMD with *K* modes and *I* iterations requires O(IKTlogT). Entropy calculation and clustering introduce only linear overhead and do not affect inference-time capacity.


** **

Online computation.

Let *d* denote hidden dimension and *N* the number of factors. Temporal encoding via LSTM and attention scales as $O(Td^{2})$. Dynamic adjacency construction requires $O(N^{2}d)$, while mix-hop propagation costs $O(L_{g}K_{\max}Ed)$, reducing to O(Nkd) under sparsification. Factor-level self-attention introduces an additional $O(N^{2}d)$ term.

Overall online complexity is $O\left(Td^{2}\right)+O\left(N^{2}d\right)+O\left(L_{g}K_{\max}Ed\right)$, with parameter growth scaling as $O\left(d^{2}\right)+O\left(Nd\right)$.


** **

Practical deployment.

Since decomposition and embedding are cached offline, inference complexity is governed by temporal encoding and factor interaction. In practical financial forecasting settings, where *N* is relatively small and the sequence length is fixed (e.g., seven steps), the model scales linearly in sequence length and near-linearly in factor size. Empirically, inference latency is comparable to standard LSTM-based models, making the framework suitable for daily-frequency forecasting without requiring specialized hardware.

## 4. Experiment

### 4.1. Dataset Descriptions and Preprocessing

For missing terms in both datasets (see Table 2), spline interpolation was applied within each training window to obtain smooth estimates consistent with the temporal dependency structure. All interpolation procedures were conducted using only information available up to the prediction time, thereby preventing look-ahead bias.

Min–max normalization was performed using parameters computed exclusively from the training set, and the same transformation was applied to validation and test sets to ensure strict separation across data partitions.


** **

Keyword Construction.

Behavioral variables are constructed from the Baidu Search Index, which provides consistent search-based attention measures throughout the sample period. Using a single platform avoids potential cross-platform measurement heterogeneity.

Keywords are selected based on economic relevance and market participation characteristics, and are systematically grouped into the following two categories:

(1) Direct price-related terms(e.g., “copper futures”, “copper futures price”), reflecting explicit trading and pricing interest;
(2) Commodity identity terms (e.g., “copper”, “steel”), capturing broader market attention and potential cross-commodity linkage effects.

Both Chinese and English equivalents are included to account for heterogeneous search behavior.

### 4.2. Hyperparameter Tuning and Overfitting Mitigation

To mitigate overfitting risks, we restrict the hyperparameter search space to a small number of key parameters with the most significant impact on performance. In practice, we primarily tune the decomposition level in the VMD-based multi-scale construction.

Other hyperparameters, including the learning rate, number of layers, hidden dimension, and model structure, follow standard configurations widely adopted in prior time-series forecasting studies, with only minimal adjustments based on validation performance.

We employ early stopping to prevent overfitting and reduce selection bias. This controlled tuning strategy ensures that the effective model capacity remains well regularized despite the modular design of the proposed framework.

### 4.3. Evaluation Metrics

In evaluating predictive performance, we employ three standard regression metrics: root mean square error (RMSE), mean absolute error (MAE), and the coefficient of determination ($R^{2}$). All evaluation metrics are computed on the out-of-sample test set, which accounts for 30% of the total sample, to assess genuine forecasting performance rather than in-sample fit. The forecasting task is formulated as a multi-step prediction problem with forecast horizons h=1,2,3. For each horizon *h*, the model generates a prediction ${\hat{y}}_{t+h}$ based only on information available up to time *t*. The predicted value is then compared with the realized price at time t+h. Let $M_{h}$ denote the number of forecast observations in the test set for horizon *h*. The evaluation metrics for each forecast horizon are defined as follows:

(12) $$\begin{array}{cc} {\mathrm{RMSE}}_{h} & =\sqrt{\frac{1}{M_{h}}\sum_{i=1}^{M_{h}}{\left(y_{i+h}-{\hat{y}}_{i+h}\right)}^{2}},\\ {\mathrm{MAE}}_{h} & =\frac{1}{M_{h}}\sum_{i=1}^{M_{h}}\left|y_{i+h}-{\hat{y}}_{i+h}\right|,\\ R_{h}^{2} & =1-\frac{\sum_{i=1}^{M_{h}}{\left(y_{i+h}-{\hat{y}}_{i+h}\right)}^{2}}{\sum_{i=1}^{M_{h}}{\left(y_{i+h}-\bar{y}\right)}^{2}}. \end{array}$$

Here, $M_{h}$ represents the number of prediction points in the out-of-sample test period for horizon *h*, and $\bar{y}$ denotes the sample mean of the realized values in the corresponding test set. RMSE penalizes large deviations more heavily due to the squared error term, while MAE provides a scale-consistent measure that is less sensitive to outliers. The $R^{2}$ statistic reflects the proportion of variance in the realized prices explained by the forecasts in the out-of-sample period, serving as a normalized measure of predictive accuracy.

### 4.4. Experimental Results

In this section, we present a series of experiments designed to benchmark the performance of the proposed model against several state-of-the-art baselines across two distinct datasets. The hyperparameter for the best result of the proposed model is shown in Table 3.

Among these baselines, Informer [24] improves computational efficiency through sparse attention, Autoformer [25] introduces trend-seasonal decomposition, TimesNet [41] uses frequency-domain block convolution to enhance local temporal modeling, and DTSformer [26] considers two-stage attention across variables and time. In addition, a Naive baseline is included, where the copper futures price of the previous day is directly used as the prediction for the current day.

#### 4.4.1. Experiment 1: Comparative Evaluation of Benchmark Models

**Figure 4:**
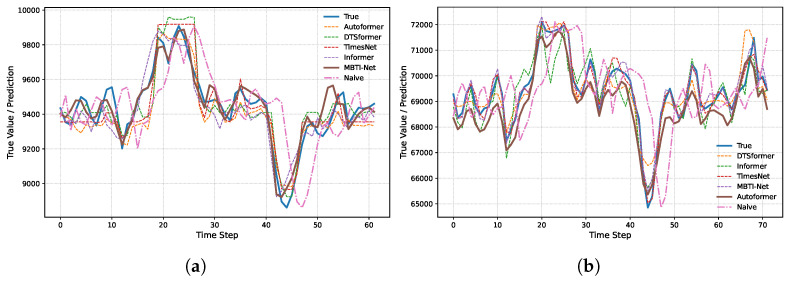
(**a**) LME dataset; (**b**) SHFE dataset. Out-of-sample 3-step-ahead (h = 3) forecasting results on the test set. The figure compares the predicted values of the proposed method and benchmark models against the actual observations.

We conducted a comprehensive evaluation of MBTI-Net against both advanced transformer-based architectures and a persistence-based Naive baseline, as summarized in Table 4, Figure 4a,b. The results consistently demonstrate the superiority of MBTI-Net across both datasets.

On the LME dataset, MBTI-Net achieves an MAE of 53.10 and an RMSE of 72.39, corresponding to reductions of 29.2% and 29.6%, respectively, compared with the strongest deep learning baseline (Autoformer). Although the Naive model exhibits competitive performance (RMSE = 134.42), MBTI-Net reduces RMSE by 46.1% and MAE by 39.3% relative to this persistence-based benchmark. The resulting $R^{2}$ of 0.991 is the highest among all evaluated models, indicating superior explanatory capability.

On the more volatile SHFE dataset, MBTI-Net attains an MAE of 149.44 and an RMSE of 202.70. Compared with the best-performing baseline (TimesNet), the reductions reach 45.2% in MAE and 44.5% in RMSE. Even relative to the Naive baseline (RMSE = 330.73), MBTI-Net achieves a substantial RMSE reduction of 38.7%. The corresponding $R^{2}$ value of 0.996 further demonstrates its strong capacity to capture the underlying dynamics of highly fluctuating market data.

#### 4.4.2. Experiment 2: Ablation Analysis of Core Architectural Innovations in MBTI-Net

As shown in Table 5, we conduct an ablation study to evaluate the contribution of each module within MBTI-Net.

Removing the Complexity-Aware VMD Reconstructionleads to a noticeable decline in forecasting accuracy on both datasets. On LME, MAE increases from 55.27 to 71.84, while on SHFE, MAE rises from 153.82 to 258.41. These results suggest that the decomposition-based reconstruction contributes to modeling non-stationary structures in the series, providing enhanced feature representations for forecasting.

Excluding behavior-induced signals results in further performance deterioration, particularly on SHFE. This finding indicates that incorporating behavioral information as an explanatory feature helps to capture additional market dynamics, particularly in volatile conditions. When the Behavior-Triggered Dynamic Factor Interaction module is removed, MAE on SHFE increases from 153.82 to 1123.59, and the corresponding $R^{2}$ decreases from 0.992 to 0.821. This substantial change implies that modeling dynamic cross-factor interactions plays a critical role in capturing complex dependencies in market behavior, beyond traditional modeling approaches. Eliminating the Hierarchical Temporal Fusion module also leads to consistent performance degradation across datasets, suggesting that multi-scale temporal aggregation provides complementary structural information, which enhances the model’s ability to handle long-term dependencies beyond single-scale representations.

#### 4.4.3. Experiment 3: Comparison Between Index-Based and Complexity-Aware VMD Grouping

**Figure 5:**
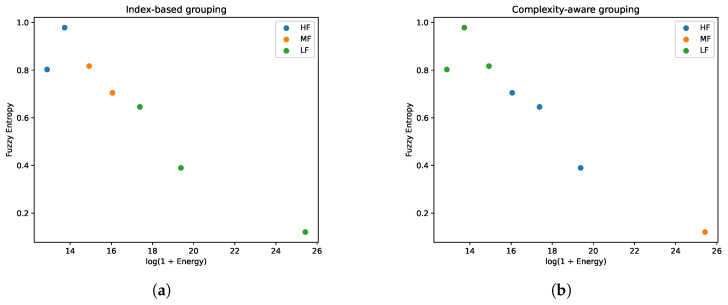
(**a**) Index-based grouping; (**b**) complexity-aware grouping. IMF distribution in the $\log(1+E_{k})$–FuzzyEn feature space.

**Figure 6:**
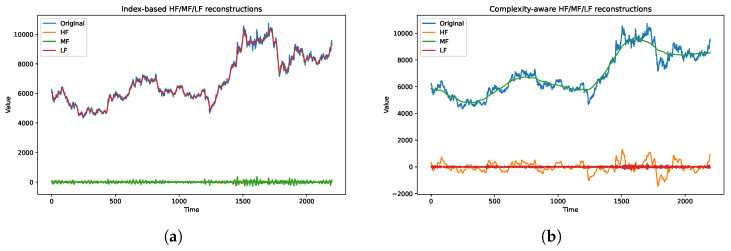
(**a**) Index-based reconstruction; (**b**) complexity-aware reconstruction. Comparison of reconstructed HF/MF/LF components.

Figure 5a and Figure 6a report the results of the conventional index-based IMF grouping. In the log(1+E)–FuzzyEn feature space, substantial overlap is observed among HF, MF, and LF modes, suggesting that the decomposition index does not strictly correspond to intrinsic frequency characteristics. The reconstructed components reflect this overlap: the LF component retains high-frequency fluctuations, while the MF and HF components exhibit limited amplitude differentiation.

Figure 5b and Figure 6b present the complexity-aware grouping results. The IMFs form more distinct clusters in the energy–entropy space, with LF, MF, and HF modes occupying separable regions. The reconstructed components display clearer scale separation, where LF captures low-frequency trends, MF represents intermediate oscillations, and HF isolates short-term variations. This separation provides a structured multi-scale representation for subsequent forecasting.

#### 4.4.4. Experiment 4: Comparative Evaluation and Statistical Significance Analysis

**Figure 7:**
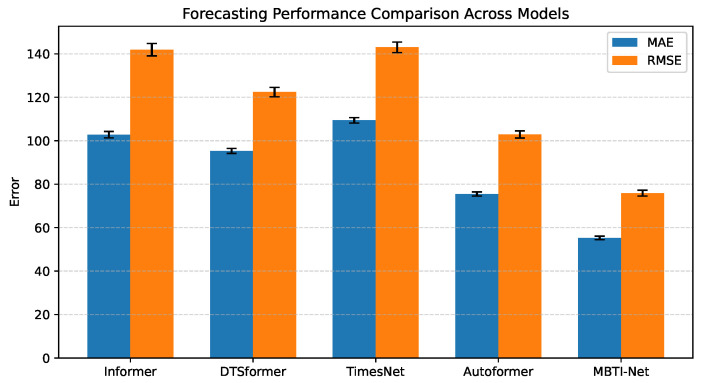
Forecasting performance of benchmark models with mean and standard deviation on the LME dataset.

Table 6 and Figure 7 report forecasting performance under a multi-origin rolling evaluation protocol. For each forecasting origin, the model is trained and evaluated across 10 independent runs with different random initialization seeds, while keeping the data splits fixed. The reported results correspond to the mean and standard deviation across these runs. Performance is assessed using MAE, RMSE, 95% confidence intervals (CIs), and Diebold–Mariano (DM) tests relative to MBTI-Net. Among the baseline models, Autoformer achieves the lowest error (MAE 75.5±0.96, RMSE 102.9±1.63), followed by DTSformer (MAE 95.3±1.12). Informer and TimesNet exhibit higher error levels across both metrics.

MBTI-Net attains the lowest overall error, with MAE 55.3±0.81 and RMSE 75.9±1.34. The corresponding 95% CI for MAE is [54.8,55.8], indicating limited variation across repeated runs. All DM *p*-values relative to MBTI-Net are below 0.05, suggesting that the observed error differentials are unlikely to be attributable to random variation under the adopted evaluation protocol.

### 4.5. Independent Evaluation of VB-ReVIN

To rigorously evaluate the standalone contribution of VB-ReVIN, we conduct controlled experiments to explicitly disentangle its effect from graph interaction and behavior-triggered structural modulation. Two structural settings are considered: (i) a graph-free backbone, where both dynamic graph interaction and behavior-triggered modulation are removed, and (ii) a graph-only configuration, where graph interaction is retained but behavioral modulation is disabled. These settings allow us to assess whether performance gains arise from normalization itself rather than from architectural enhancements.

Under each setting, we compare five normalization strategies: (1) no normalization; (2) ReVIN; (3) adaptive scaling [42], which dynamically updates normalization statistics via time-adaptive mean and variance estimation without explicit regime modeling or reversible affine transformation; (4) regime-switch normalization, which performs regime-wise standardization based on volatility states but does not include learnable affine reconstruction; and (5) VB-ReVIN (ours), which integrates volatility-aware regime conditioning with group- and regime-dependent reversible affine transformation.

For fairness, regime quantile thresholds and all normalization statistics are estimated exclusively on the training set and fixed for validation and testing. Volatility regimes are computed causally using only past returns within the input window, ensuring the absence of look-ahead bias.

Table 7 reports the results. Under the graph-free setting, VB-ReVIN consistently achieves the lowest MAE and RMSE across both datasets, demonstrating that its performance improvement does not rely on graph interaction. On LME, MAE decreases from 74.516 (ReVIN) to 64.381, with $R^{2}$ increasing from 0.980 to 0.987. On SHFE, MAE decreases from 232.774 to 198.623, while $R^{2}$ improves from 0.983 to 0.994. Notably, VB-ReVIN also outperforms adaptive scaling and regime-switch normalization, indicating that volatility-conditioned reversible affine transformation provides additional benefits beyond drift adaptation or regime-wise standardization alone.

Under the graph-only configuration, VB-ReVIN again yields the best performance across all metrics. On LME, MAE decreases from 65.973 (ReVIN) to 60.204, and on SHFE from 210.744 to 170.933. The consistent gains under both structural settings suggest that the contribution of VB-ReVIN is orthogonal to graph modeling and primarily stems from improved distribution stabilization under volatility regime shifts.

### 4.6. Regime-Dependent Analysis

To evaluate whether the behavioral module provides state-dependent predictive value, we conduct a volatility-based regime analysis to test robustness under changing market conditions (Table 8).


** **

Regime Definition.

Market regimes are defined using rolling realized volatility of the corresponding futures index. The test set is divided into the following:

- Low-volatility regime: volatility ≤ median;
- High-volatility regime: volatility > median.

All models are evaluated out of sample without retraining.

MBTI-Net consistently outperforms its ablated version in both regimes, indicating stable generalization. Notably, the relative gain increases under high volatility. Since high-volatility periods correspond to stronger non-stationarity, the amplified improvement suggests that the behavioral module captures uncertainty-sensitive information rather than merely increasing model capacity.

## 5. Conclusions

This study proposes a unified forecasting framework called MBTI-Net to model and predict the conditional mean of copper futures prices in the presence of non-stationarity, multi-scale dynamics, and behavioral influences. MBTI-Net integrates complexity-aware variational mode decomposition, multi-scale temporal modeling, and heterogeneous behavioral indicators, enabling it to capture intrinsic market dynamics and exogenous information flows that are often overlooked by traditional models.

A key finding of this study is that behaviorally driven exogenous signals (including search intensity, online attention, and mobile activity) exhibit incremental predictive value, serving as proxy indicators for information demand, trading participation, and emerging macroeconomic concerns. On datasets from LME and SHFE, these behavioral variables improve forecast accuracy, particularly during periods characterized by elevated uncertainty or sentiment-driven fluctuations. The Hierarchical Temporal Fusion and behavior-triggered interaction modules effectively integrate external signals with endogenous time patterns, achieving substantial reductions in mean absolute error (MAE) and root mean square error (RMSE) relative to competitive baseline models.

Nevertheless, several limitations should be acknowledged. First, behavioral attention is proxied using the Baidu Search Index as a unified platform across both markets. While this ensures methodological consistency and avoids cross-platform measurement discrepancies, it may limit regional representativeness, particularly for the LME market, where platforms such as Google Trends may better reflect European investor attention. Cross-platform behavioral heterogeneity is not modeled in the current framework.

Second, although the model delivers robust predictive improvements, the behavioral indicators remain correlational. The framework prioritizes forecasting performance rather than formal identification of economic transmission mechanisms. Consequently, the causal channels through which attention dynamics affect commodity prices—such as liquidity adjustments, risk reallocation, or volatility spillovers—are not explicitly disentangled.

Future research could extend this framework by integrating multiple search platforms to examine cross-regional behavioral heterogeneity and comparing platform-specific attention measures. Incorporating alternative data sources—such as social media sentiment, institutional positioning data, or real-time news analytics—may further enrich behavioral representations. Moreover, exploring structural or causal modeling approaches to formally identify the transmission channels between behavioral signals and commodity price dynamics represents a promising direction.

## Figures and Tables

**Table 1:** Summarized categories of copper futures price forecasting models in literature.

Category	Techniques	Representative Studies and Key Insights
Statistical Models	ARIMA, SARIMA, SARIMAX, MIDAS, etc.	[31] Traditional time-series models with variants that incorporate exogenous variables and mixed-frequency data.
Machine Learning Models	SVM, RF, GBRT, KNN, RT, ELM, etc.	[2,31] Data-driven methods suitable for structured features-based forecasting tasks.
Deep Learning	RBFNN, LSTM, GRU, Transformer, etc.	[22,23,32,33,34] Capable of modeling nonlinear and long-term dependencies in copper futures price series.
Hybrid and Optimized Models	PSO-LSTM, PSO-ELM, GA-LSTM-MIDAS, etc.	[28,35] Combine deep learning, signal decomposition, and metaheuristic optimization to improve prediction accuracy.
Decomposition and Frequency Analysis	Wavelet, VMD, NMEPS, Chaos Theory, etc.	[9,29,30] Extract multi-scale components or patterns from raw prices for enhanced forecasting robustness.

Note: Autoregressive integrated moving average (ARIMA), seasonal autoregressive integrated moving average (SARIMA), seasonal autoregressive integrated moving average with exogenous variables (SARIMAX), mixed data sampling (MIDAS), support vector machine (SVM), random forest (RF), gradient boosting regression tree (GBRT), K-nearest neighbors (KNNs), regression tree (RT), extreme learning machine (ELM), radial basis function neural network (RBFNN), long short-term memory (LSTM), gated recurrent unit (GRU), particle swarm optimization (PSO), genetic algorithm (GA), variational mode decomposition (VMD), nonparametric multi-scale entropy-based prediction system (NMEPS).

**Table 2:** Data card for the copper futures and Baidu Index datasets.

Item	Description
Time span	5 January 2015 to 16 April 2024
Sampling frequency	Daily observations
Total sample size	2257 timestamps after preprocessing
Target variable	Close price
Data source	Wind Financial Terminal website (https://www.wind.com.cn/portal/zh/WFT/index.html, accessed on 8 March 2026); data collected via web scraping.
Keyword list	{“copper futures”, “copper price”, “copper”, “steel futures”, “steel”, etc.}
Region	National-level Baidu Index
Platform	Aggregated from PC and mobile search volumes
Alignment strategy	Aligned by calendar date; missing dates forward-filled
Missing-value handling	Spline interpolation followed by forward-filling
Outlier treatment	Winsorization at 1st/99th percentiles
Normalization	Min–max scaling applied to all features
Train/test split	70%/30% chronological split
Validation protocol	Last 10% of training portion for validation
Early stopping	Patience = 5 epochs based on validation loss
Hyperparameter tuning	Grid search over learning rate, batch size, and hidden units

**Table 3:** Best training hyperparameters for the proposed model on two datasets.

Training Parameter	LME Dataset	SHFE Dataset
Batch size	16	16
Dropout rate	0.1	0.2
Learning rate	0.002	0.0035
Lookback window size	7	7
Prediction time step size	3	3

**Table 4:** Comparisons among the overall accuracies of different models on both LME and SHFE datasets.

Model	LME Dataset			SHFE Dataset		
	MAE	RMSE	$R^{2}$	MAE	RMSE	$R^{2}$
Naive	87.538	134.422	0.983	259.638	330.727	0.978
DTSformer	94.711	121.223	0.975	468.672	665.086	0.966
Informer	100.048	127.865	0.972	1037.14	1415.86	0.847
TimesNet	109.011	142.070	0.965	272.729	365.298	0.989
Autoformer	75.0327	102.751	0.982	702.457	780.502	0.953
**MBTI-Net**	**53.103**	**72.385**	**0.991**	**149.444**	**202.698**	**0.996**

**Table 5:** Ablation study on the key innovative components of MBTI-Net across two datasets. Each row removes one innovation module to assess its contribution to predictive performance.

Model Variant	LME Dataset			SHFE Dataset		
	MAE	RMSE	$R^{2}$	MAE	RMSE	$R^{2}$
w/o Complexity-Aware VMD Reconstruction	71.84	98.12	0.984	258.41	349.55	0.987
w/o Behavior-Induced Signals (Baidu Index)	89.72	118.03	0.977	661.84	742.51	0.951
w/o Behavior-Triggered Dynamic Factor Interaction	104.35	131.66	0.970	1123.59	1493.27	0.821
w/o Hierarchical Temporal Fusion	96.28	124.90	0.973	514.72	702.18	0.955
Full MBTI-Net	**55.27**	**75.91**	**0.987**	**153.82**	**208.77**	**0.992**

**Table 6:** Forecasting performance comparison across models. All results are reported as mean ± std over 10 independent runs using a multi-origin rolling evaluation protocol. To assess statistical significance, 95% confidence intervals (CI) and Diebold–Mariano (DM) *p*-values are included. Lower values indicate better performance.Bold values indicate the best performance.

Model	MAE (Mean ± Std)	RMSE (Mean ± Std)	95% CI (MAE)	DM *p*-Value vs. MBTI-Net
Informer	102.8±1.47	141.9±2.85	[101.9,103.7]	0.044
DTSformer	95.3±1.12	122.4±2.18	[94.6,96.0]	0.027
TimesNet	109.4±1.23	143.0±2.44	[108.7,110.2]	0.071
Autoformer	75.5±0.96	102.9±1.63	[74.9,76.1]	0.029
**MBTI-Net**	55.3±0.81	75.9±1.34	[54.8,55.8]	–

**Table 7:** Controlled evaluation of VB-ReVIN under graph-free and graph-only settings on LME and SHFE datasets.

Model Variant	LME Dataset			SHFE Dataset		
	MAE	RMSE	$R^{2}$	MAE	RMSE	$R^{2}$
*Baseline (graph-free)*						
None	78.442	108.371	0.979	265.388	352.107	0.980
Adaptive scaling	72.893	100.654	0.983	238.541	315.733	0.976
Regime-switch normalization	70.772	97.215	0.984	229.906	303.104	0.962
ReVIN	74.516	103.208	0.980	232.774	311.865	0.983
**VB-ReVIN (ours)**	**64.381**	**89.742**	**0.987**	**198.623**	**267.881**	**0.994**
*Graph-only (without behavior-triggered modulation)*						
None	72.185	99.452	0.964	255.537	335.804	0.951
Adaptive scaling	68.812	93.776	0.977	225.362	295.492	0.974
Regime-switch normalization	74.947	102.541	0.960	195.718	252.381	0.995
ReVIN	65.973	90.388	0.985	210.744	278.615	0.985
**VB-ReVIN (ours)**	**60.204**	**83.615**	**0.990**	**170.933**	**228.114**	**0.996**

**Table 8:** Regime-split performance of MBTI-Net and its ablated variant.

Model	Low-Vol MAE	High-Vol MAE	Low-Vol RMSE	High-Vol RMSE
MBTI-Net (w/o Behav.)	65.42	78.37	89.15	105.28
MBTI-Net (Full)	**50.91**	**55.62**	**69.84**	**75.11**
Relative Gain (%)	22.2%	**29.0%**	21.6%	**28.7%**

## Data Availability

The raw datasets used in this study were obtained from publicly accessible official websites, including the London Metal Exchange (LME) and the Shanghai Futures Exchange (SHFE), as well as from Wind (https://www.wind.com.cn/portal/zh/WFT/index.html, accessed on 10 March 2024). The processed datasets constructed for the empirical analysis, together with the source code used to generate the results, are available from the corresponding author upon reasonable request. The authors are committed to facilitating replication and academic transparency.
